# Negative impact of the hypopnea index or duration increase after a non-frame work surgery in patients with very severe obstructive sleep apnea

**DOI:** 10.1038/s41598-022-06293-6

**Published:** 2022-02-10

**Authors:** Ethan I. Huang, Shu-Yi Huang, Yu-Ching Lin, Chieh-Mo Lin, Chin-Kuo Lin, Ying-Chih Huang, Chia-Yu Hsu, Jian-An Su

**Affiliations:** 1 Department of Otolaryngology, Chang Gung Memorial Hospital, Chiayi, 61363 Taiwan, ROC; 2 Sleep Center, Chang Gung Memorial Hospital, Chiayi, 61363 Taiwan, ROC; 3 Department of Medicine, School of Medicine, Chang Gung University, Taoyuan, 33302 Taiwan, ROC; 4 Division of Pulmonary and Critical Care Medicine, Chang Gung Memorial Hospital, Chiayi, 61363 Taiwan, ROC; 5 Department of Nursing, Chang Gung University of Science and Technology, Chiayi, 61363 Taiwan, ROC; 6 Department of Respiratory Care, Chang Gung University of Science and Technology, Chiayi, 61363 Taiwan, ROC; 7 Graduate Institute of Clinical Medical Sciences, College of Medicine, Chang Gung University, Taoyuan, 33302 Taiwan, ROC; 8 Department of Neurology, Chang Gung Memorial Hospital, Chiayi, 61363 Taiwan, ROC; 9 Department of Psychiatry, Chang Gung Memorial Hospital, Chiayi, 61363 Taiwan, ROC

**Keywords:** Surgery, Respiratory tract diseases

## Abstract

A non-framework surgery could change the postoperative components of breathing disturbances and increase the frequency or duration of hypopnea in patients with very severe obstructive sleep apnea (OSA). Either an increase of hypopnea index, which increases apnea–hypopnea index (AHI), or an increase of its duration raises the concern of worsening the oxygen desaturation and so morbidity and mortality associated with OSA. It is unclear how the oxygen saturation would change in those having increased frequency or duration of hypopneas after the surgery. Here in 17 patients with AHI ≥ 60 events/h, having increased frequency or duration of hypopneas after the non-framework surgery, the results show that the surgery improved oxygen saturation by reducing obstructive-apnea index (36.1 events/h) and duration (8.6 s/event), despite it increased hypopnea index (16.8 events/h) and duration (9.8 s/event). The surgery improved the average of the mean oxyhemoglobin saturation of pulse oximetry (SpO2) by 2.8% (toward a ceiling mean of 94.3%), mean minimal SpO2 by 7.5%, and mean desaturation by 5%. The results suggest sufficient apnea reduction and shift from apnea to hypopnea may mask the negative impact of the increase of hypopnea index or duration and improve postoperative mean SpO2, minimal SpO2, and mean desaturation.

## Introduction

Hypopnea is a part of AHI, which serves as a disease- and severity-defining metric for OSA^1^. In our earlier works, the hypopnea ratio in AHI may be increased after a non-framework surgery^2^ by decreasing apneas or converting apneas into hypopneas in patients with very severe OSA^3,4^. Here a non-framework surgery is defined as a procedure that does not involve the skeletal framework of the upper airway, i.e., maxilla and mandible. An increase of hypopnea ratio or duration raises the concern of worsening the oxygen desaturation associated with the disease.

Oxygen desaturation is associated with event duration^5–8^. For both components (of apnea and hypopnea), OSA patients with long average apnea–hypopnea duration had worse mean oxygen saturation percentage than the patients with short average duration^5^. For apneas, patients with a long mean apnea duration had worse blood oxygen levels and sleep parameters^6^. For hypopneas, increased hypopnea duration may worsen SpO2 desaturation, but hypopneas were reported to lead to less severe SpO2 desaturation compared to obstructive apneas^7^. A similar study by Yılmaz Durmaz and Gunes reported that longer mean hypopnea duration was related to lower mean oxygen saturation but was not associated with an oxygen desaturation event below 85%^8^. The common ground is that the worse oxygen desaturation can come from more events or longer duration of hypopnea. The effect of hypopneas on oxygen desaturation is smaller than that of obstructive apneas.

The non-framework surgery^2^ we modified from Friedman’s^9–11^ reduced AHI and apneas but may increase the frequency or duration of hypopneas^2–4^. In patients with very severe OSA, although our earlier reports showed improvement of oxygen saturation after the surgery^3,4^, it is unclear whether this resulted from those without increased frequency or duration of hypopneas. There is a concern how the oxygen saturation would change in those having increased frequency or duration of hypopneas after the surgery. The aim of the study is to investigate whether hypopnea index or duration increase after the surgery might worsen oxygen saturation. Here we screened out patients with very severe OSA with increased frequency or duration of hypopneas after the surgery by multiplying hypopnea index (events/h) with mean hypopnea duration (s). Among them, we compared hypopnea index, mean hypopnea duration, obstructive-apnea index, mean obstructive-apnea duration, mean SpO2, minimal SpO2, and mean desaturation before and after the surgery. The results show that the reduction of frequency or duration of obstructive apneas may mask the negative effect of increased frequency or duration of hypopneas and improved the above oxygen saturation parameters.

## Materials and methods

From 2015 to 2020, adult patients with very severe OSA that met these criteria were enrolled:

- Age 20 or older
- AHI equals to or over 60 events/h
- Refusal or unsuccessful of continuous positive airway pressure (CPAP)
- Received a one-stage multi-level sleep surgery with the modified Z-palatoplasty (ZPP) performed with one-layer closure and open partial tongue-base glossectomy^2^
- Underwent polysomnographies (PSGs) before and after the surgery with needed recordings, including mean SpO2, minimal SpO2, and mean desaturation
- Increased frequency or duration of hypopneas after the surgery.

We illustrated and detailed the surgery in the earlier works^2,3^. Each PSG was carried out overnight in the level 1 sleep laboratory of the authors’ tertiary referral hospital by a certified technician and interpreted by a certified sleep physician. In a PSG, a hypopnea is defined as a decrease in airflow ≥ 30% for at least 10 s accompanied by either electroencephalography (EEG) signs of arousal or by a 4% or greater decrease in oxygen saturation^12^. An apnea is defined as the complete cessation of airflow for at least 10 s^12^. Increased frequency or duration of hypopneas is defined as having an increase in the product of hypopnea index (events/h) and mean hypopnea duration (s).

To further understand the impact of one sleep parameter, we conducted a paired t-test for each of AHI, hypopnea index, mean hypopnea duration, obstructive-apnea index, and mean obstructive-apnea duration. We scatter plotted and calculated the product of hypopnea index and mean hypopnea duration and that of obstructive-apnea index and mean duration of obstructive apnea, with a paired t-test to analyze the differences against no difference after the surgery. Then we scatter plotted and conducted a paired t-test for each of mean SpO2, minimal SpO2, and mean desaturation before and after the surgery. A Lilliefors test was performed to test the normality of differences before and after the surgery for all of the parameters mentioned above. A p value < 0.05 indicated a statistical significance.

The statistical examinations were conducted in Matlab 9.4.0.813654 (MathWorks, Natick, Massachusetts, U.S.A.).

### Ethical statements

The Institutional Review Board (IRB) of Chang Gung Medical Foundation, Taiwan, approved the study methods and protocols (IRB number: 202001198B0). We performed the study under Good Clinical Practice and the laws and regulations. As a retrospective cohort study, the IRB approved the waiver of the participants' consent.

## Results

Fifteen men and two women with very severe OSA aged between 25 and 63 years were enrolled in this work. The average body mass index (BMI) was 28.9 with a standard deviation of 3.6 kg/m^2^. A PSG was performed about 6 months (192 ± 78 days) after the surgery, when the anatomy was considered stable (as the period of 3 to 6 months was adopted in the literature, e.g., see^13,14^). The Lilliefors tests showed that the differences before and after the surgery for all of the tested parameters were normally distributed, except for the product difference of hypopnea index and mean hypopnea duration. Among these 17 patients, the surgery reduced AHI from 73.7 ± 9.2 (a standard deviation) to 45.3 ± 15.1 events/h, p < 0.001. The mean AHI reduction was 28.4 events/h, with a 95% confidence interval of 19.7 to 37.1 events/h. Although the mean BMI decreased 1.2 kg/m^2^ (p = 0.044), with a 95% confidence interval of 0.04 to 2.42 kg/m^2^, BMI change was not statistically correlated with AHI reduction (r = 0.129, p = 0.621).

The preoperative hypopnea index ranged from 0.6 to 34.1 events/h. The postoperative hypopnea index ranged from 6.4 to 53 events/h. A paired t-test showed hypopnea index increased from a mean of 14.4 ± 10.9 events/h to 31.2 ± 15.4 events/h, p < 0.001. The mean increase of hypopnea index was 16.9 events/h, with a 95% confidence interval of 8.9 to 24.9 events/h. The preoperative mean hypopnea duration ranged from 16.3 to 31.5 s. The postoperative mean hypopnea duration ranged from 21 to 68.7 s. A paired t-test showed mean hypopnea duration increased from 24.0 ± 6.1 s to 33.8 ± 12.6 s, p = 0.011. The mean increase of hypopnea duration was 9.7 s, with a 95% confidence interval of 2.5 to 17.0 s.

The preoperative obstructive-apnea index ranged from 16.2 to 73 events/h. The postoperative obstructive-apnea index ranged from 0 to 50.8 events/h. A paired t-test showed obstructive-apnea index reduced from a mean of 46.3 ± 15.2 events/h to 10.2 ± 14.0 events/h, p < 0.001. The mean reduction of apnea index was 36.0 events/h, with a 95% confidence interval of 26.7 to 45.3 events/h. The preoperative mean obstructive-apnea duration ranged from 18.5 to 47.8 s. The postoperative mean obstructive-apnea duration ranged from 0 to 40.2 s. A paired t-test showed mean obstructive-apnea duration decreased from 29.7 ± 9.3 s to 21.1 ± 11.6 s, p = 0.0015. The mean reduction of apnea duration was 8.5 s, with a 95% confidence interval of 3.8 to 13.3 s.

**Figure 1:**
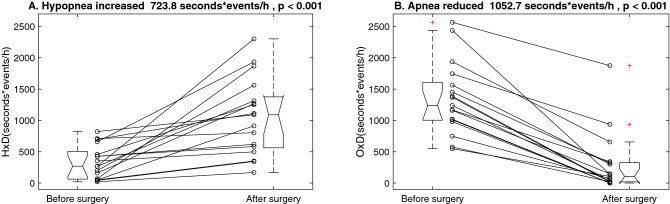
Individual product of (**A**) hypopnea index and mean hypopnea duration and (**B**) obstructive-apnea index and mean obstructive-apnea duration before and after the surgery. The surgery reduced the product on obstructive apnea but increased that on hypopnea.

Figure 1 shows the individual product of hypopnea index and mean hypopnea duration and that of obstructive-apnea index and mean obstructive-apnea duration before and after the surgery. The surgery increased the mean product on hypopnea from 334 ± 265 to 1058 ± 612 s*events/h. The average increase of the mean product on hypopnea was 724 s*events/h, with a 95% confidence interval of 404 to 1043 s*events/h. It reduced the mean product on obstructive apnea from 1348 ± 574 to 295 ± 482 s*events/h. There is a probable floor effect on this reduction (panel B). The average reduction of the mean product on obstructive apnea was 1053 s*events/h, with a 95% confidence interval of 835 to 1270 s*events/h.

**Figure 2:**
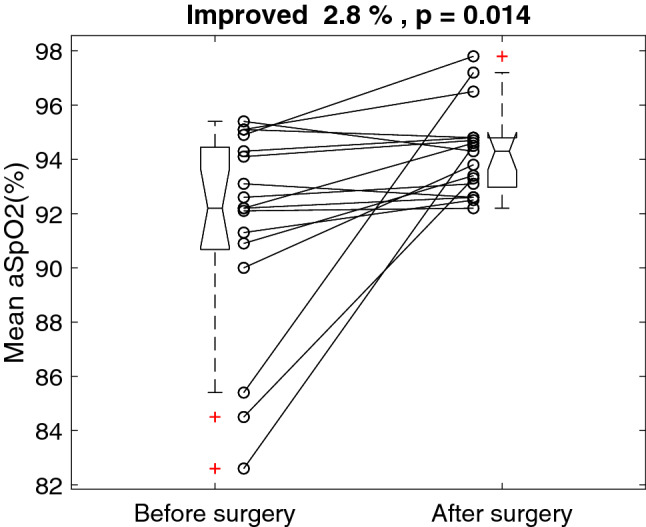
Individual mean oxyhemoglobin saturation of pulse oximetry (SpO2) before and after the surgery.

Figure 2 shows the individual mean SpO2 before and after the surgery. The surgery improved the average of the mean SpO2 by 2.8% (with a ceiling effect) from 91.5 ± 3.9 to 94.3 ± 1.6%, p = 0.014. The mean SpO2 improved 2.8%, with a 95% confidence interval of 0.6 to 4.9%

**Figure 3:**
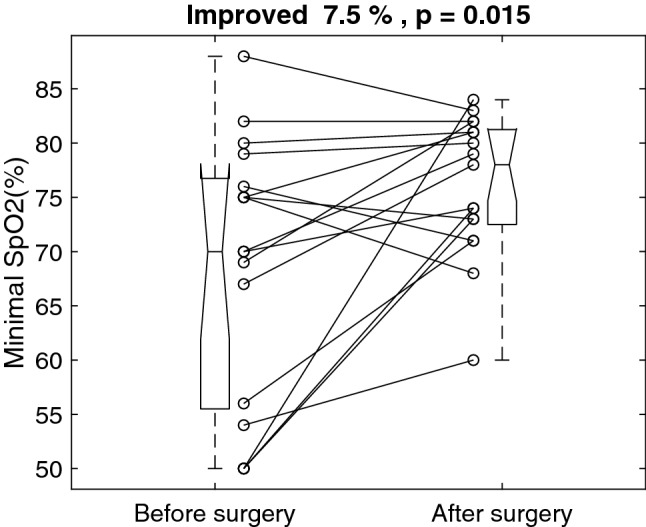
Individual minimal oxyhemoglobin saturation of pulse oximetry (SpO2) before and after the surgery.

Figure 3 shows the individual minimal SpO2 before and after the surgery. The surgery improved mean minimal SpO2 by 7.5% from 68.6 ± 12.2 to 76.1 ± 6.4%, p = 0.015. The mean minimal SpO2 improved 7.5%, with a 95% confidence interval of 1.7 to 13.4%

**Figure 4:**
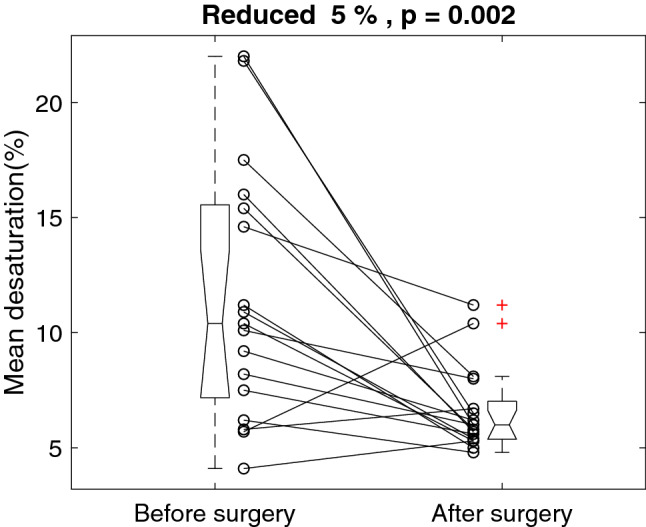
Individual mean desaturation before and after the surgery.

Figure 4 shows the individual mean desaturation before and after the surgery. The surgery reduced mean desaturation by 5% from 11.6 ± 5.5 to 6.6 ± 1.8%, p = 0.002. The mean desaturation reduced 5.0%, with a 95% confidence interval of 2.1 to 7.9%

## Discussion

In patients with very severe OSA with AHI ≥ 60 events/h and having increased frequency or duration of hypopneas after the non-framework surgery, the results show that the surgery improved oxygen saturation by reducing obstructive-apnea index (36.1 events/h) and duration (8.6 s/event), despite it increased hypopnea index (16.8 events/h) and duration (9.8 s/event). The surgery improved the average of the mean SpO2 by 2.8% (with a ceiling effect to a postoperative mean of 94.3%), mean minimal SpO2 by 7.5%, and mean desaturation by 5%. There are very few detailed reports of hypopnea in patients with very severe OSA. The sample size of 17 in this study is small, although it is larger than 6 in Walker’s^15^, 9 in Jacobowitz’s^16^, 10 in Mickelson’s^17^, and 11 in Vilaseca’s^18^ for patients with very severe OSA. That limits the generalizability of the results. It needs future studies with a larger sample size to verify the observation. The results suggest that a sufficient shift from complete (apnea) to partial (hypopnea) cessation of airflow may mask the negative impact of the increase of hypopnea index or duration.

Respiratory disturbances can have unique characteristics even though the numbers are similar^8^. The AHI, which comprises the number of apneas and hypopneas, incorporates no information of the duration of these events. It is unlikely that an event lasting 10 s is physiologically equivalent to an event lasting 2 or 3 min^19^. Omission of event duration is another weakness of the AHI as a disease- or severity-defining metric for OSA^19^. One patient in the present study had a worse AHI from 62.6 to 77.6 after the surgery. But for the components, his obstructive-apnea index improved from 38.6 to 31.9 events/h, mean obstructive-apnea duration from 45.2 to 29.4 s, mean SpO2 from 85.4 to 97.2%, minimal SpO2 from 50 to 84%, and mean desaturation from 21.8 to 6.5%, despite the increase of AHI.

Deeper desaturations or longer breathing cessations may have more severe consequences than shorter and shallower ones^20^. Pulmonary artery hemodynamics (mean pulmonary artery pressure and right atrial pressure) are associated with duration of nocturnal desaturation (percentage of sleep time spent with oxygen saturation < 90%) but not AHI^21^. If severity was measured by the product of duration and depth of the individual obstruction event, the severity of individual obstruction events (not the AHI number) is related to the increased mortality rate in severe obstructive sleep apnea^22^. These support that AHI may weigh less on disease severity than desaturation and that an adequate shift from apnea to hypopnea may reduce the severity of the disease.

## Conclusions

The frequency or duration of hypopnea could increase in patients with very severe OSA with AHI ≥ 60 events/h after a non-framework surgery. But the results showed that sufficient apnea reduction and the shift from apnea to hypopnea might mask the negative impact of the increase of hypopnea index or duration and improve postoperative mean SpO2, minimal SpO2, and mean desaturation.
